# A moiré deflectometer for antimatter

**DOI:** 10.1038/ncomms5538

**Published:** 2014-07-28

**Authors:** S. Aghion, O. Ahlén, C. Amsler, A. Ariga, T. Ariga, A. S. Belov, K. Berggren, G. Bonomi, P. Bräunig, J. Bremer, R. S. Brusa, L. Cabaret, C. Canali, R. Caravita, F. Castelli, G. Cerchiari, S. Cialdi, D. Comparat, G. Consolati, H. Derking, S. Di Domizio, L. Di Noto, M. Doser, A. Dudarev, A. Ereditato, R. Ferragut, A. Fontana, P. Genova, M. Giammarchi, A. Gligorova, S. N. Gninenko, S. Haider, T. Huse, E. Jordan, L. V. Jørgensen, T. Kaltenbacher, J. Kawada, A. Kellerbauer, M. Kimura, A. Knecht, D. Krasnický, V. Lagomarsino, S. Lehner, A. Magnani, C. Malbrunot, S. Mariazzi, V. A. Matveev, F. Moia, G. Nebbia, P. Nédélec, M. K. Oberthaler, N. Pacifico, V. Petràček, C. Pistillo, F. Prelz, M. Prevedelli, C. Regenfus, C. Riccardi, O. Røhne, A. Rotondi, H. Sandaker, P. Scampoli, J. Storey, M.A. Subieta Vasquez, M. Špaček, G. Testera, R. Vaccarone, E. Widmann, S. Zavatarelli, J. Zmeskal

**Affiliations:** 1 Politecnico di Milano, Piazza Leonardo da Vinci 32, 20133 Milan, Italy; 2 Istituto Nazionale di Fisica Nucleare, Sez. di Milano, Via Celoria 16, 20133 Milan, Italy; 3 Physics Department, European Organisation for Nuclear Research, 1211 Geneva 23, Switzerland; 4 Laboratory for High Energy Physics, Albert Einstein Center for Fundamental Physics, University of Bern, 3012 Bern, Switzerland; 5 Institute for Nuclear Research of the Russian Academy of Sciences, Moscow 117312, Russia; 6 Department of Mechanical and Industrial Engineering, University of Brescia, Via Branze 38, 25133 Brescia, Italy; 7 Istituto Nazionale di Fisica Nucleare, Sez. di Pavia, Via Agostino Bassi 6, 27100 Pavia, Italy; 8 Kirchhoff Institute for Physics, Heidelberg University, Im Neuenheimer Feld 227, 69120 Heidelberg, Germany; 9 Department of Physics, University of Trento and TIFPA-INFN, Via Sommarive 14, 38123 Povo, Trento, Italy; 10 Laboratoire Aimé Cotton, CNRS, University of Paris-Sud, ENS Cachan, Bâtiment 505, Campus d’Orsay, 91405 Orsay, France; 11 Physics Institute, University of Zurich, Winterthurerstrasse 190, 8057 Zurich, Switzerland; 12 Department of Physics, University of Milan, Via Celoria 16, 20133 Milan, Italy; 13 Max Planck Institute for Nuclear Physics, Saupfercheckweg 1, 69117 Heidelberg, Germany; 14 Istituto Nazionale di Fisica Nucleare, Sez. di Genova, Via Dodecaneso 33, 16146 Genoa, Italy; 15 Institute of Physics and Technology, University of Bergen, Allégaten 55, 5007 Bergen, Norway; 16 Department of Physics, University of Oslo, Sem Sælandsvei 24, 0371 Oslo, Norway; 17 Department of Physics, University of Genoa, Via Dodecaneso 33, 16146 Genoa, Italy; 18 Stefan Meyer Institute for Subatomic Physics, Austrian Academy of Sciences, Boltzmanngasse 3, 1090 Vienna, Austria; 19 Department of Physics, University of Pavia, Via Bassi 6, 27100 Pavia, Italy; 20 Joint Institute for Nuclear Research, Dubna 141980, Russia; 21 Istituto Nazionale di Fisica Nucleare, Sez. di Padova, Via Marzolo 8, 35131 Padua, Italy; 22 Institute of Nuclear Physics of Lyon, CNRS/IN2P3, University Lyon 1, 69622 Villeurbanne, France; 23 Czech Technical University in Prague, FNSPE, Břehová 7, 11519 Prague 1, Czech Republic; 24 Department of Physics and INFN Bologna, University of Bologna, Via Irnerio 46, 40126 Bologna, Italy; 25 Department of Physics, University of Napoli Federico II, Via Cinthia, 80126 Naples, Italy

## Abstract

The precise measurement of forces is one way to obtain deep insight into the fundamental interactions present in nature. In the context of neutral antimatter, the gravitational interaction is of high interest, potentially revealing new forces that violate the weak equivalence principle. Here we report on a successful extension of a tool from atom optics—the moiré deflectometer—for a measurement of the acceleration of slow antiprotons. The setup consists of two identical transmission gratings and a spatially resolving emulsion detector for antiproton annihilations. Absolute referencing of the observed antimatter pattern with a photon pattern experiencing no deflection allows the direct inference of forces present. The concept is also straightforwardly applicable to antihydrogen measurements as pursued by the AEgIS collaboration. The combination of these very different techniques from high energy and atomic physics opens a very promising route to the direct detection of the gravitational acceleration of neutral antimatter.

## Abstract (web summary)

Measuring forces on antimatter is vital to testing our understanding of fundamental physics. Towards this aim, Aghion *et al.* present a method to measure the deflection of antiprotons based on an atom optical tool, the moiré deflectometer, which could be extended to future antihydrogen gravity measurements.

## Graphical abstract

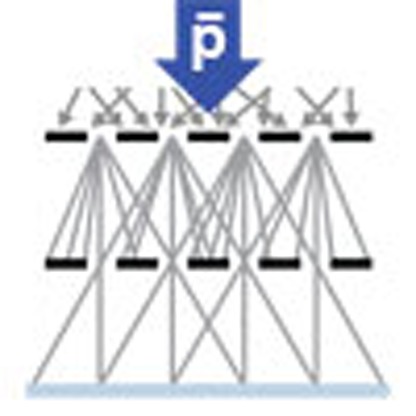

The precise measurement of forces between objects gives deep insight into the fundamental interactions and symmetries of nature. A paradigm example is the comparison of the motion of matter in the gravitational field, testing with high precision that the acceleration is material-independent, that is, the weak equivalence principle^1^,^2^,^3^,^4^. Although indirect experimental evidence suggests that the weak equivalence principle also holds for antimatter^5^,^6^,^7^, a direct observation for antimatter is still missing. First attempts in this direction have recently been reported by the ALPHA collaboration^8^, who used the release of antihydrogen from a magnetic trap to exclude the absolute value of the gravitational acceleration of antihydrogen to be 100 times larger than for matter. An alternative approach is followed by the GBAR collaboration^9^, which is based on sympathetic cooling of positive antihydrogen ions and their subsequent photodetachment. One of the specified goals of the AEgIS collaboration (antihydrogen experiment: gravity, interferometry, spectroscopy) is the direct detection of the gravitational acceleration using an antihydrogen beam^10^,^11^ combined with a moiré deflectometer^12^, a device with high sensitivity for acceleration measurements.

Here, we present the successful realization of such a device for antiprotons. This has been achieved using slow antiprotons from the Antiproton Decelerator (AD) at CERN, the technology of emulsion detectors developed for recent high-energy neutrino experiments^13^ and a novel referencing method employing Talbot–Lau interferometry^14^,^15^ with light. The observation is consistent with a force at the 500 aN level acting on the antiprotons. This demonstration is an important prerequisite for future studies of the gravitational acceleration of antimatter building on an antihydrogen beam.

## Results

### Moiré deflectometer

**Figure 1 f1:**
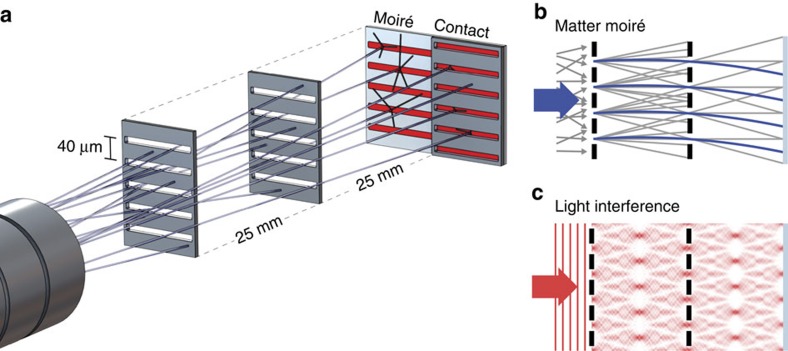
Moiré deflectometer for antiprotons. (**a**) A divergent antiproton beam impinges on two subsequent gratings that restrict the transmitted particles to well-defined trajectories. This leads to a shadow fringe pattern as indicated in **b**, which is shifted in the presence of a force (blue trajectories). Finally, the antiprotons are detected with a spatially resolving emulsion detector. To infer the force, the shifted position of the moiré pattern has to be compared with the expected pattern without force. (**c**) This is achieved using light and near-field interference, the shift of which is negligible. A grating in direct contact with the emulsion is used to reference the antimatter and the light measurements.

The principle used in the experiment reported here is visualized in Fig. 1a. A divergent beam of antiprotons enters the moiré setup consisting of three equally spaced elements: two gratings and a spatially resolving emulsion detector. The two gratings with periodicity *d* define the classical trajectories leading to a fringe pattern with the same periodicity at the position of the detector. If the transit time of the particles through the device is known, absolute force measurements are possible by employing Newton’s second law of mechanics^16^. As indicated in Fig. 1b, the position of the moiré pattern is shifted in the presence of a force with respect to the geometric shadow by


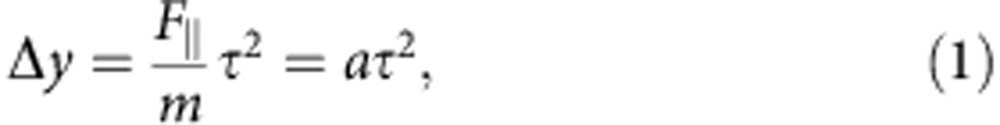


where *F*_||_ [Fig f1]represents the force component along the grating period, *m* is the inertial mass of the test particle, *a* is the acceleration and *τ* is the time of flight between the two gratings. It is important to note that the shift has two contributions. The velocity of the particle after the second grating in the direction of the acceleration is non-zero and the particle is also accelerated in the second half of the moiré deflectometer. The relevant parameter for precision measurements is the sensitivity, that is, the minimal detectable acceleration *a*_min_. This can be estimated by considering the maximal signal *S* to noise ratio possible in this scenario. Since the influence of a pattern shift is most sensitively detected at the steepest gradient of the pattern the visibility *υ*=(*S*_max_−*S*_min_)/(*S*_max_+*S*_min_) should be maximized and the periodicity minimized. The noise of the signal is intrinsically limited for classical particle sources to the shot noise which scales as 
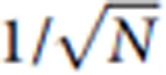
 where *N* is the number of detected particles. Consequently, the minimal detectable acceleration^12^ is given by 
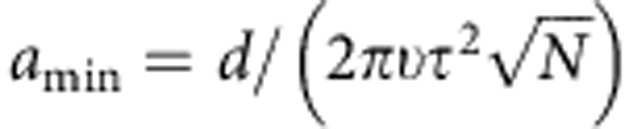
. It is important to note that this device works even for a very divergent source of particles as shown in Fig. 1a, and thus is an ideal device for the highly divergent beam of antihydrogen atoms that is expected in the AEgIS apparatus.

### Talbot–Lau interferometry with light as absolute reference

To determine the magnitude of the fringe pattern shift, knowledge of the undeflected fringe position (indicated as grey trajectories in Fig. 1b) is required. Due to the neutrality and high speed of photons, it is favourable to measure this position independently with light so that the action of forces is negligible. Unlike the case of classical particles described above, geometric paths are not applicable for visible light as diffraction at the gratings has to be taken into account. Figure 1c depicts the corresponding light field pattern where the distance between the gratings is given by the Talbot length *L*_Talbot_=2*d*^2^/*λ*. This configuration is known as Talbot–Lau interferometer^14^, which is based on the near-field Talbot effect^15^—the rephasing of the pattern in discrete distances behind a grating illuminated with light. The final pattern is not a classical distribution, but an interference pattern and coincides with the pattern of the moiré deflectometer experiencing no acceleration. Thus, light provides the required absolute zero-force reference. The only prerequisite is that the Talbot length (or a multiple integer of it) is matched to the distance between the gratings and the detector. With that, the absolute shift of the antimatter pattern can be directly accessed and systematic errors can be significantly reduced as the moiré deflectometer and Talbot–Lau interferometer use the same gratings. We would like to stress that Talbot–Lau interferometry is also possible for matter waves such as atoms and molecules^17^,^18^ if their de Broglie wavelength is long enough.

### Experimental implementation

The experiment was performed within the AEgIS apparatus designed to produce antihydrogen for a future measurement of the gravitational acceleration^10^,^19^. A beam of antiprotons with a broad energy distribution, delivered by the AD at CERN, is realized after the 5.3 MeV antiprotons are transmitted through degrader foils with a total thickness of 225 μm (170 μm of aluminium and 55 μm of silicon). The simulated distribution has a mean energy of 106 keV and a root mean squared value of about 150 keV (see Methods). After traversing a 3.6-m long tube within two homogeneous magnetic fields of 5 T and 1 T, the antiprotons enter the deflectometer. We estimate the mean de Broglie wavelength to be 8.8 × 10^−14^ m, which implies that the concept of classical paths for the trajectories of the antiprotons is applicable for our gratings with a periodicity of 40 μm.

The grating holder is compact (25 mm distance between the gratings) so that the passive stability of the relative positions between the gratings for the long measurement time of 6.5 h is ensured. The slit arrays are manufactured in silicon by reactive ion etching, leading to a 100-μm thick silicon membrane with a slit width of 12 μm and a periodicity of *d*=40 μm. Low-energy antiprotons hitting the slit array annihilate on the surface of the array and do not reach the detector. For this measurement, the final pattern, that is, the annihilation positions of antiprotons after passing two gratings, is detected by an emulsion detector. The moiré deflectometer and the annihilation detector are mounted in a vacuum chamber (10^−5^ mbar) on the extraction line of the AEgIS apparatus. After the exposure to antiprotons, the emulsion detector is removed, developed and analysed with an automatic microscope available at one of the participating institutions to determine the location of single annihilations. This facility was initially developed for the detection of neutrino-induced τ-leptons by the OPERA experiment^13^. The development of emulsion detectors for the application presented here, which involves operation in vacuum, is described in refs 20, 21.

After removal of the emulsion detector the pattern of the Talbot–Lau interferometry with light was recorded in a subsequent measurement. For this purpose, the grating holder was homogenously illuminated by an incoherent light source (red light-emitting diode with spatial diffuser). For a wavelength of *λ*=640 nm, the Talbot distance is *L*_Talbot_=2*d*^2^/*λ*≈5 mm. Thus, for our setup (*L*=25 mm), we analyse the fifth rephasing of the light waves. The light pattern was directly recorded at the plane of the emulsion with a high-resolution flatbed charge-coupled device scanner (2.7 μm resolution). To align the antiproton and light measurement in the experiment reported here, an independent spatial reference is implemented. For that purpose we installed an additional transmission grating in direct contact with the detector plane. Contact grating and moiré deflectometer (see Fig. 1a) were simultaneously illuminated: first with antiprotons and subsequently with light. In each case, the pattern behind the contact grating is a simple shadow without any force dependence, and thus can be used as a reference for alignment.

### Antimatter fringe patterns

**Figure 2:**
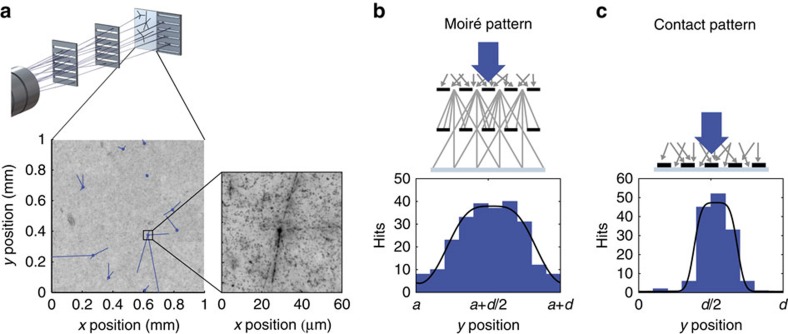
Antiproton fringe pattern. (**a**) The spatial pattern of the antiprotons (highlighted as blue tracks) as detected by the emulsion detector in an exemplary area of 1 mm^2^. The annihilation of an antiproton leads to a clear signal from which the annihilation vertex can be extracted with a precision of 2 μm by reconstruction analysing the emitted secondary particles. The image enlargement shows an exemplary annihilation star. (**b**) The fringe pattern after transmission through the moiré deflectometer setup reveals a visibility as high as (71±10) %. Since less than one antiproton is detected per lattice period, the pattern shown is obtained by binning the vertical positions modulo the extracted periodicity of the fringe pattern. The solid black line denotes the expected distribution. (**c**) The pattern behind a grating placed directly on the emulsion detector (‘contact’) is a simple shadow that is smeared out due to the finite resolution of the detection. The few background events are consistent with independently observed grating defects. This pattern is used as a reference with no force dependence since the transit time is zero. The position of the moiré fringe pattern (indicated as offset *a*) is measured using light.

With the emulsion detector, the positions of the annihilation vertices can be detected with a typical resolution of 2 μm (see Fig. 2a). The fragments produced by the annihilation of antiprotons lead to a characteristic star-shaped pattern, which can be observed with the microscope (an example is depicted in Fig. 2a). The first observation of such an annihilation star succeeded shortly after the discovery of the antiproton using emulsions^22^. This allows for very robust and high-quality particle identification, which makes this detector practically background-free. In addition, this detector can detect the arrival of antiprotons over a large area and thus is compatible with an upscaling of the grating area necessary for experiments with a divergent antihydrogen beam.

The pattern of 146 antiprotons detected for the grating in direct contact with the emulsion is depicted in Fig. 2c. The high visibility implies that the periodicity is well-defined in an area as large as 15 × 6 mm^2^ since the data collapses onto one fringe by taking the detected position modulo the extracted periodicity *d* of the pattern. To extract the periodicity, we employ the Rayleigh test^23^ that is also widely used in astronomy^24^. The periodicity *d* and the relative rotation *α* of the pattern is found by maximizing


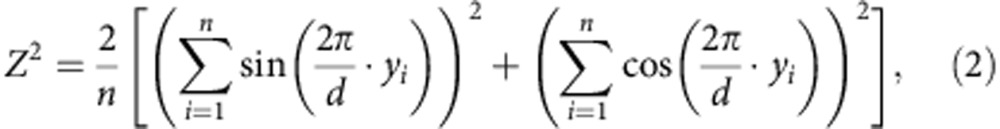


where *n* is the total number of antiprotons and *y*_*i*_=*y*′·cos *α*+*x*′·sin *α* depicts the antiproton’s projected coordinate. This leads to an inferred periodicity of 40.22±0.02 μm, which is consistent with the expected emulsion expansion of ~1% and the nominal periodicity of 40 μm. It is interesting to note that the analysed area corresponds to 368 slits and, on average, only in every second slit an antiproton is detected.

In Fig. 2b, the observed moiré pattern for antiprotons is shown. The 241 events associated with antiproton annihilations were accumulated during the 6.5-h run of the experiment. The Rayleigh tests on sub-segments of the detected patterns reveal local distortion due to the expansion/shear of the emulsion and allow the identification of regions with negligible distortion. We have restricted the areas to two-thirds of their initial size, which ensures a position uncertainty due to shear to be smaller than ±1.2 μm.

### Absolute deflection measurement

**Figure 3:**
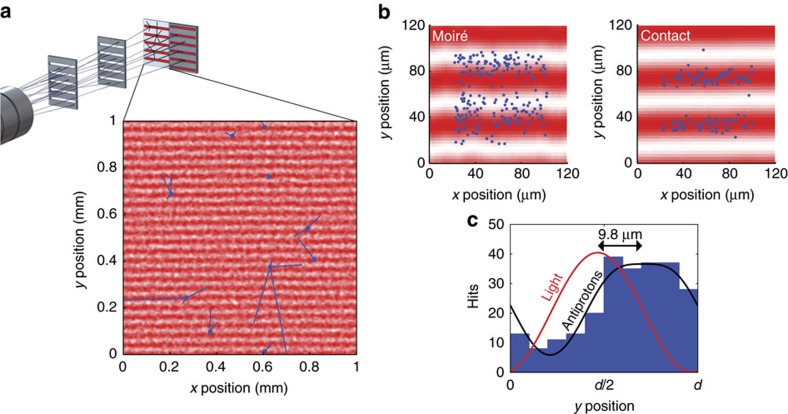
Comparison between photon and antiproton patterns. (**a**) The spatial positions of the detected antiprotons (blue dots) are compared with the subsequently recorded light pattern (measured intensity indicated by the red shading). The Talbot–Lau fringe pattern provides the zero-force reference, presented here for the same exemplary detector area with ten annihilations as in Fig. 2a. (**b**) The antiproton and light measurements are aligned by overlaying the two patterns obtained with the contact grating. The result of this procedure is visualized on the right, where the annihilation positions of all antiprotons are folded into an area of 80 × 80 μm^2^. The moiré and Talbot–Lau pattern depicted on the left, without any further alignment, can be compared to determine a shift. (**c**) The data is projected onto the *y* axis for quantitative analysis. A relative shift between moiré and Talbot–Lau pattern indicates that a force is present. The observed mean shift of 9.8 μm is consistent with a mean force of 530 aN.

To determine the absolute position of the antiproton fringe pattern (parameter *a* in Fig. 2b), we conduct a comparison with the measurement with light. The results are represented in Fig. 3a,b where the detected intensity is indicated by the red shading. The alignment is achieved by overlaying the contact patterns as depicted on the right of Fig. 3b. The moiré pattern can now be directly compared with the Talbot–Lau pattern (left of Fig. 3b) to extract a possible deflection.

For the quantitative analysis, we extract the orientation of the antimatter (Rayleigh test) and light patterns (Fourier transformation as the data is discrete in space). We find that the relative angle of the two antiproton patterns, which are 15 mm apart, deviates from the angle measured between the two corresponding light patterns by Δ*θ*=0.92±0.27 mrad.

This observation is consistent with independent systematic studies of the distortion of emulsions on this large scale^25^. It is important to realize that this angle implies an intrinsic systematic uncertainty in the determination of the relative shift between the light and antimatter patterns since one cannot know which part has undergone the deformation. Assuming that both areas of the emulsion corresponding to contact and moiré have changed the same way on the centimetre scale, that is, half of the angular deviation for each pattern, we can compare the relative positions of the antiprotons with that of the light pattern as shown in Fig. 3b. The contact patterns on the right overlay as these are direct shadows of the grating (no force dependence), while an upward shift of the antiprotons in the force sensitive moiré pattern is noticeable. For quantitative analysis, we collapse the data onto one fringe (see Fig. 3c) and deduce the relative shift of Δ*y*_mean_=9.8±0.9 μm (stat.) where the error is due to the uncertainties (one sigma) of the involved fits. Estimating a bound on the systematic uncertainties, we repeat our analysis assuming that either the contact or the moiré pattern has been changed due to the distortion. With that we find a minimal shift of Δ*y*_min_=3.7±0.9 μm (stat.) and maximal shift of Δ*y*_max_=16.4±0.9 μm (stat.) leading to a shift of Δ*y*_mean_=9.8±0.9 μm (stat.)±6.4 μm (syst.).

## Discussion

The observed shift of the moiré pattern is consistent with a force acting on the antiprotons. With the assumption of a mean velocity of *v*=4.5 × 10^6^ ms^−1^ implying a transit time of *τ*=5.6 ns, we find a mean force of *F*=530±50 aN (stat.)±350 aN (syst.).

**Figure 4:**
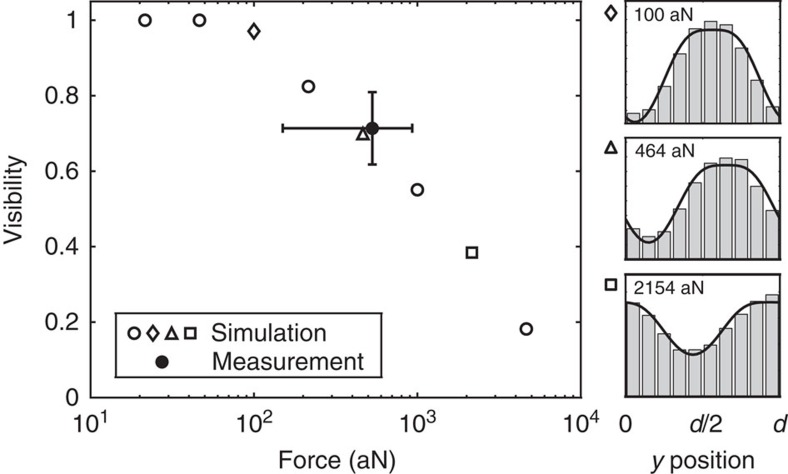
Monte Carlo simulation. A detailed simulation study based on the expected energy distribution of the antiprotons (see Methods) shows the visibility for increasingly large forces. As the observed pattern in the presence of a force is an ensemble of differently shifted patterns corresponding to different transit times *τ* the visibility consequently decreases. The measured fringe pattern exhibits a visibility of (71±10) % and is consistent with the result of this simulation. The error bar on the measured visibility is determined via resampling; the error bar on the measured force includes the systematic error bound and the one sigma statistical error bound. The observed high visibility excludes that the fringe pattern is shifted by more than one period and sets an upper limit for a force present without the necessity of referencing.

It is important to note that the mere observation of a pattern sets an upper bound for the force being present. The impinging antiproton beam has a very broad velocity distribution due to the degrading process in the foils. Thus, in the case that a force is present, the experimentally observed moiré pattern is an ensemble of differently shifted patterns corresponding to the transit times *τ* for different velocities. The results of a simulation of the performance of the moiré deflectometer are depicted in Fig. 4 and clearly reveal how the visibility vanishes for increasingly large forces (a force of 10 fN reduces the visibility below *υ*=10%). The observed visibility of 71% is consistent with a mean force of ~500 aN. The visibility of the antiproton moiré pattern on its own (not relying on additional referencing) is an independent consistency check that the observed pattern is indeed shifted due to a force. Additionally with the observed high visibility of the moiré pattern, we exclude the possibility that the force has shifted the pattern by more than one period (see Fig. 4).

The measured force could arise from a Lorentz force either caused by an electric field of ~33 V cm^−1^ in direction of the grating period or a magnetic field component of ~7.4 G perpendicular to the grating period and antiproton direction. The latter is compatible with the measured magnetic field of ~10 G at the position of the deflectometer due to the fringe field of the trapping region and stray fields of neighbouring experiments in the AD zone.

The results presented are a crucial step towards the direct detection of gravitational acceleration of antihydrogen with the AEgIS experiment. Its concept is based on the formation of excited antihydrogen through the charge exchange reaction of electromagnetically trapped antiprotons with bunched Rydberg positronium. The resulting dipole moments of the antihydrogen atoms in a weak electric field allow their subsequent acceleration with electric field gradients, thus forming a beam towards the moiré deflectometer. The measurement of the antihydrogen’s arrival position is realized by detection of the annihilation of its antiproton—thus using techniques presented here.

It is important to note that the expected absolute shift of the antihydrogen pattern due to gravity is comparable to the one observed in the current experiment. Although the gravitational force acting on antihydrogen is 10 orders of magnitude smaller than the sensitivity level reached with the presented small prototype deflectometer, the resolution of the setup can be simply improved by scaling up the deflectometer and the detector. A detailed discussion of the expected performance can be found in refs 10, 19. The main improvement is achieved by increasing the transit time *τ* (see equation (1)). Using a beam of antihydrogen atoms with a significantly lower velocity of ~500 ms^−1^ and a distance of 1 m between the gratings (this experiment *v*=4.5 × 10^6^ ms^−1^ and *L*=25 mm) will improve the sensitivity by 11 orders of magnitude (eight orders of magnitude due to slower velocity and three orders of magnitude due to increased length of the device), thus allowing the application of this technique to direct measurements of the gravitational force with antihydrogen.

High resolution is a prerequisite for the successful direct detection of the gravitational acceleration of antimatter. For absolute measurements, the sensitivity is the relevant parameter, which is ultimately limited by the intrinsic shot noise due to the detection of single atoms. Since the sensitivity scales with 1/_det_ (*N*_det_ representing the number of detected particles) increasing the length of the moiré setup implies a similar expansion of the transverse dimensions to keep the throughput, and thus the flux, high. We have already successfully produced high-quality gratings with a transverse extent as large as 100 mm. The currently limiting systematic errors due to the distortion of the emulsion can be overcome by referencing the antihydrogen pattern directly to an *in situ*-realized light pattern, by employing emulsions on a glass substrate instead of the plastic used for this measurement or by the use of photomasks^25^.

## Methods

### Orientation of the antiproton pattern

**Figure 5:**
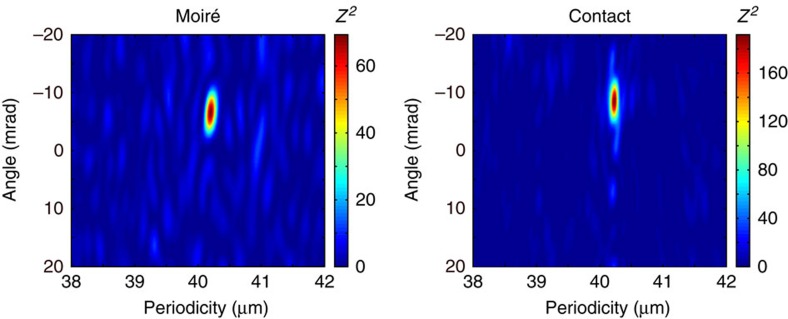
Rayleigh test. The results of the Rayleigh test applied on the antiproton data of the moiré deflectometer and the contact grating show unambiguous maxima from which orientation and periodicity of the patterns are extracted.

Collapsing the detected annihilation events to a single histogram as shown in Fig. 2 relies on the accurate determination of the periodicity as well as the angular orientation of the two dimensional fringe pattern. For this reason, we conduct the Rayleigh test given by equation (2) for different angles and periodicities and the results are depicted in Fig. 5 for the moiré and the contact pattern. The maximum is well-defined so that periodicity and angular orientation can be extracted for further analysis. The performance of the Rayleigh test has been tested with simulated data sets.

### Velocity distribution

The kinetic energy of the antiprotons reaching the deflectometer is estimated with a simulation based on Geant4 (ref. 26)^26^. All the materials interposed along the beamline, between the AD antiproton beam (initial kinetic energy of 5.3 MeV) and the deflectometer, as well as the magnetic field and geometry of the AEgIS apparatus, are taken into account.

**Figure 6:**
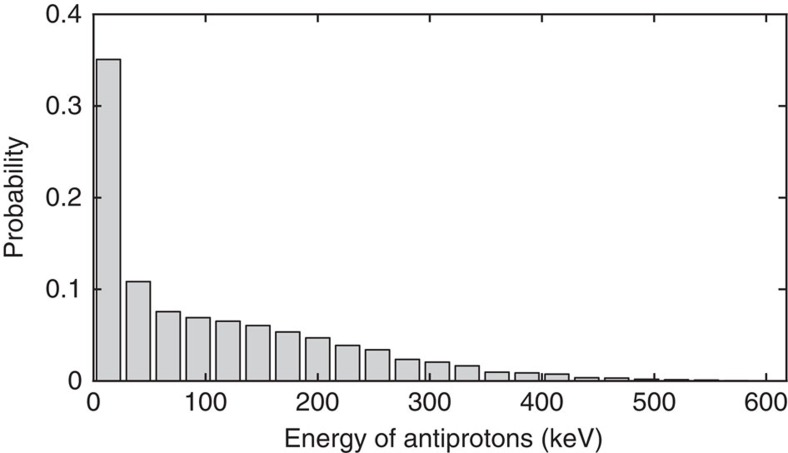
Simulated energy distribution. A Monte Carlo simulation based on the Geant4 toolkit provides an estimate of the kinetic energy distribution of the antiprotons reaching the moiré deflectometer. This calculation takes into account the degrading foil system, the magnetic field and the geometry of the AEgIS apparatus.

The input parameters of the Monte Carlo simulation are matched to meet the experimentally observed best antiproton trapping efficiency. This measurement was performed by counting the number of trapped antiprotons for various thicknesses of an aluminium degrader placed upstream of the antiproton trap. The thickness of the degrader foils used to slow down the antiprotons was selected in order to maximize the number of antiprotons with a kinetic energy lower than 10 keV that enter the antiproton trap and was set to 55 μm of silicon and 170 μm of aluminium. An additional foil of titanium (2 μm) is placed at the exit of the 1 T magnet. The energy distribution at the position of the deflectometer is depicted in Fig. 6. It is very broad and thus can be employed for setting limits on the maximum force present by analysing the visibility of the moiré pattern.

## Author contributions

All authors contributed equally to the work presented.

## Additional information

**How to cite this article:** Aghion, S. *et al.* A moiré deflectometer for antimatter. *Nat. Commun.* 5:4538 doi: 10.1038/ncomms5538 (2014).
